# Association of increased Treg and Th17 with pathogenesis of moyamoya disease

**DOI:** 10.1038/s41598-017-03278-8

**Published:** 2017-06-08

**Authors:** Leihua Weng, Xiang Cao, Lijuan Han, Haoran Zhao, Shuwei Qiu, Yaping Yan, Xiaoying Wang, Xiangyan Chen, Weihong Zheng, Xin Xu, Yuanyuan Gao, Yan Chen, Jie Li, Yongbo Yang, Yun Xu

**Affiliations:** 1 Department of Neurology and Neurosurgery, Drum Tower Hospital of Medical School and the State Key Laboratory of Pharmaceutical Biotechnology, Nanjing University, Nanjing, P. R. China; 2 Departments of Neurology, Affiliated Zhongshan Hospital of Xiamen University, 201 Hubinnan Road, Xiamen, 361004 China; 3 Jiangsu Key Laboratory for Molecular Medicine, Medical School of Nanjing University, Nanjing, P. R. China; 4 Jiangsu Province Stroke Center for Diagnosis and Therapy, Nanjing, P. R. China; 5 College of Cife Sciences, Shanxi Normal University, Xian, P. R. China; 6 Departments of Neurology, Massachusetts General Hospital, Harvard Medical School, Charlestown, MA USA; 7 Departments of Medicine and Therapeutics, Chinese University of Hong Kong, Shatin, Hong Kong SAR China; 8 Department of Neurology, Affiliated Yixing People’s Hospital of Jiangsu University, Yixing, Jiangsu P. R. China

## Abstract

Immuno-inflammation has been shown to play a pivotal role in the pathogenesis of moyamoya disease (MMD). However, how did circulating Treg/Th17 cells involve in MMD patients remains unclear. 26 MMD, 21 atherothrombotic stroke, and 32 healthy controls were enrolled in this study. MMD patients have a significantly higher percentage of circulating Treg and Th17 cells as well as their dominantly secreting cytokines than other groups (*P* < 0.0001), whereas no difference was found in the ratio of Treg/Th17 between patients in MMD and atherothrombotic stroke group or control subjects (*P* = 0.244). However, the increased Treg in MMD patients which were enriched with FrIII Treg cells had deficient suppressive functions (*P* = 0.0017) compared to healthy volunteers. There was a positive correlation between Treg or TGF-β and MMD Suzuki’s stage. And the level of circulating Treg was as an independent factor associated with MMD stage. Besides, TGF-β was also correlated with the increased expression of VEGF in MMD patients. Our findings indicated an important involvement of circulating Treg in the pathogenic development of MMD and TGF-β in Treg induced VEGF.

## Introduction

Moyamoya disease (MMD) is a chronic steno-occlusive cerebrovascular disorder characterized by progressive stenosis of the terminal internal carotid artery (ICA) and/or its proximal branches, typically accompanied by an abnormally thin and fragile collateral vascular network at the base of the brain developed to compensate as the blood flow decreases in the affected area^1^. Accumulating studies showed that inflammation and autoimmune responses may be involved in the development of MMD^2–4^. Currently, imbalance of two unique CD4^+^T helper (Th) cell subsets, Treg and Th17 cells, has been implicated in the pathogenesis of a wide spectrum of autoimmune and inflammatory diseases^5, 6^. Moreover, different Treg subsets with distinct functions were identified recently^6–8^. For example, CD4^+^CD25^+^FoxP3^low^CD45RA^−^Tregs cells also named as Fraction (Fr) III Treg potent effector-Th cells capabilities^9^. However, whether Treg/Th17 cells are involved in the pathogenic development of MMD remains to be explored.

Vascular endothelial growth factor (VEGF) plays an essential role in the formation and maintenance of moyamoya vessels in MMD patients^10^. Interestingly, Treg have been shown to promoting angiogenesis via secreting VEGFA in response to hypoxia^11^. Besides, Treg-produced transforming growth factor (TGF)-β1 could induce production of VEGF and stimulate subsequent angiogenesis^12^. Nonetheless, whether Treg/Th17 contribute to the production of VEGF and subsequent angiogenesis in development of MMD was unknown.

Therefore, we would like to investigate here whether imbalance of Treg/Th17 is associated with the course of MMD, and if so whether it contributes to abnormal angiogenesis in MMD through regulation of VEGF signaling. Our results will clarify the importance of Treg/Th17s in the pathogenesis of MMD, thus providing a potential therapeutic target for MMD patients.

## Materials and Methods

### Patients

This study was approved by the ethics committee of the affiliated Drum Tower Hospital, Nanjing University Medical School, and all experiments were performed in accordance with relevant guidelines and regulations. Twenty-six inpatients diagnosed with definitive MMD by DSA according to the diagnostic criteria^13^ were enrolled from the Department of Neurology and Neurosurgery of Drum Tower Hospital between 2013 and 2014. After a detailed explanation of the study procedures, all participants or their guardians signed an informed consent form before the study. Patients who met any of the following criteria were excluded: 1) diagnosed with Quasi-moyamoya disease; 2) active intracranial hemorrhage including intracerebral hemorrhage, intraventricular hemorrhage, and subarachnoid hemorrhage; 3) patients were not suitable for DSA or MRI; 4) patients with severe infection and other disease which could influence inflammation and autoimmune responses. For controls we recruited twenty-one atherothrombotic stroke patients diagnosed by guidelines for diagnosis and therapy on Chinese patients with acute ischemic stroke^14^ and thirty-two age-matched healthy volunteers in this research. To minimize the effects of stroke on inflammation and autoimmune responses, heparinized whole blood samples of MMD and atherothrombotic stroke patients were collected at 48 ± 16.3 days after stroke which is believed as the recovery phase following ischemic stroke.

### Flow cytometry

To investigate the changes of Treg and Th17 cells in MMD patients, flow cytometry was used to measure the percentage of Th17 and Treg cells. Briefly, peripheral blood mononuclear cells (PBMCs) were isolated from the fresh blood by Ficoll gradient centrifugation. For Th17 cells, the isolated PBMCs were pre-treated with phorbol 12-myristate-13-acetate (PMA, 25 ng/ml), ionomycin (50 µg/ml) and brefeldin-A(BFA, 10 µg/ml, Enzo Life Sciences, Farmingdale, USA) at 37 °C for 4 h, then were collected and surface-stained with antibodies (eBiosicence, Frankfurk, Germany) at room temperature (RT) for 15 min, followed by fixed and permeabilized with the transcription factor staining buffer set kit according to the manufacturer’s protocol (eBioscience), then intracellular-stained with IL-17 antibody (eBioscience) for 30 min. Treg and its subtypes were incubated with surface antibodies at RT for 15 min. After fixed and permeabilized, cells were stained with Foxp3 antibody (eBiosicence) at RT for 30 min. Isotype controls were used in parallel. CD4^+^CD25^+^Foxp3^+^ cells were considered as Treg cells while CD3^+^CD8^−^IL-17^+^ cells were defined as Th17 cells. Cells were measured using flow cytometry (BD bioscience, San Diego, CA, USA). Data were analyzed using Flowjo software (Tree Star, Ashland, OR).

### Cytometric Bead Array (CBA) and enzyme-linked immunosorbent assay (ELISA) assay

Next, to evaluate the role of Treg/Th17-driven inflammation, serum cytokines from Treg/Th17, Th1/Th2, and other inflammatory and angiogenenic factors, including interleukin (IL)-2, IL-4, IL-6, IL-10, IL-17, IL-23, tumor necrosis factor (TNF)-α, interferon (INF)-γ, VEGF, intercellular adhesion molecule (ICAM)-1 and vascular cell adhesion molecule (VCAM) were measured by Cytometric Bead Array (CBA, human inflammation kit, BD Biosciences) according to the manufacturer’s instructions. TGF-β and high-mobility group protein (HMGB)-1 were measured by a commercially available ELISA (R&D Systems, Minneapolis, MN) according to the manufacturer’s instructions.

### Cell purification and Treg suppressive assay

To determine whether the Treg from MMD have immunosuppressive capacity, the proliferation effect of T cells was measured by flow cytometry. Treg cells were isolated using human CD4^+^CD25^+^Treg Isolation Kit (MiltenyiBiotec, Inc., Auburn, CA) according to the manufacturer’s instructions. The CD4^+^CD25^−^ fraction was defined as effector T (Teff) cells. The Treg suppressive assay was conducted as described before^15^. In brief, 5 * 10^4^ carboxyfluorescein diacetate succinimidyl ester (CFSE)-labeled Teff cells (2.5 µM) per well were plated and cultured in a 24-well plate with Treg cells (Teff/Treg = 1:1) in the presence of CD3/CD28 antibodies (5 ng/ml), TGF-β (10 ng/ml) and IL-2 (100 IU/ml) for 96 h. The Teff cells were harvested and evaluated by flow cytometry. Cultured CD4^+^CD25^−^ cells were taken as control group. The data were analyzed using Modfit software (Verity Software House 2.0, Top-sham, ME, USA).

### Statistical Analysis

All samples and standards were assayed in triplicate. We used SPSS version 19.0 (SPSS Inc, New York, USA) to perform the statistical analyses. Shapiro-Wilk test was used to measure normality of data. Normal continuous variables were presented as mean ± S.D, while non-normal continuous variables were displayed as median and quartiles. Categorical variables were presented as percentages. For normal variables, Student’s t-test and one-way ANOVA were used to comparison between two groups and more than two groups, respectively. For non-normal variables, comparison between two groups and more than two groups was statistically evaluated by Mann-Whitney U-test and Kruskal-Wallis test, respectively. A multiple linear regression analysis was conducted for investigating the impact of Treg, Th17, TGF-β, IL-10 and IL-17 on the serum VEGF. Correlation of the inflammatory and angiogenic factors with the severity of MMD was evaluated by Spearman correlation coefficient (r). The predictive value of serum cytokines on the severity of MMD was tested by binary of logistic regression analysis. A *P* value < 0.05 was considered statistically significant.

## Results

### Clinical features of patients

Between Jan 2013 and Dec 2014, a total of 26 consecutive patients with MMD (10 females and 16 males), 21 patients with atherothrombotic stroke (8 females and 13 males) and 32 age-matched healthy volunteers (19 females and 13 males) as controls were enrolled into our cohort. General clinical features of control group, MMD and atherothrombotic stroke patients are summarized in Table 1. The common initial presentations in MMD patients was ischemic stroke in our study (46.15%) followed by cerebral hemorrhage (26.92%), subarachnoid hemorrhage (23.08%) and TIA (3.85%, Table 2). The majority of patients were presented at Suzuki Angiographic stage 4 and 5 (53.84% and 23.08%, respectively) and 92.3% of patients had bilateral lesions. Besides, there was no significant difference in leukocyte count and C-reactive protein (CRP)-levels among MMD, atherothrombotic stroke and controls (data not shown).

**Table 1:** Clinical characteristics of control group, MMD and atherothrombotic stroke.

	Normal (N = 32)	MMD (N = 26)	Stroke (N = 21)
Age	38.50 ± 0.6720	44.08 ± 1.860	64.62 ± 8.077
Gender (F/M)	19/13	10/16	8/13
Hypertension	0/32	8/26	16/21
Diabetes Mellitus	0/32	3/26	7/21
Autoimmune Disease	0/32	0/26	0/21

**Table 2:** Initial symptom and Suzuki’s angiographic stage of MMD.

Variable	Numbers (N = 26)
Initial symptom	
TIA	1 (3.85%)
Ischemia	12 (46.15%)
Cerebral hemorrhage	7 (26.92%)
SAH	6 (23.08%)
Bilateral lesion	24 (92.3%)
**Suzuki’s angiographic stage**	
1	0
2	2 (7.69%)
3	1 (3.84%)
4	14 (53.84%)
5	6 (23.08%)
6	3 (11.54%)

SAH: subarachnoid hemorrhage.

### Increased peripheral Treg and Th17 in MMD patients

Compared to control, the serum protein level of several inflammatory molecules including HMGB-1, ICAM-1 and VCAM in MMD patients was strikingly increased by 92.32% [57.000 (34.000, 154.000) vs. 27.000 (13.500, 61.000), P < 0.05], 31.66% [38.070 (31.750, 42.760) vs. 28.080 (21.280, 34.905), *P* < 0.01] and 51.5% [158.135 (130.560, 211.360) vs. 103.975 (85.435, 135.810), *P* < 0.001], respectively. (Supplementary Fig. 1) These findings indicate that peripheral inflammatory responses were induced in MMD patients.

**Figure 1:**
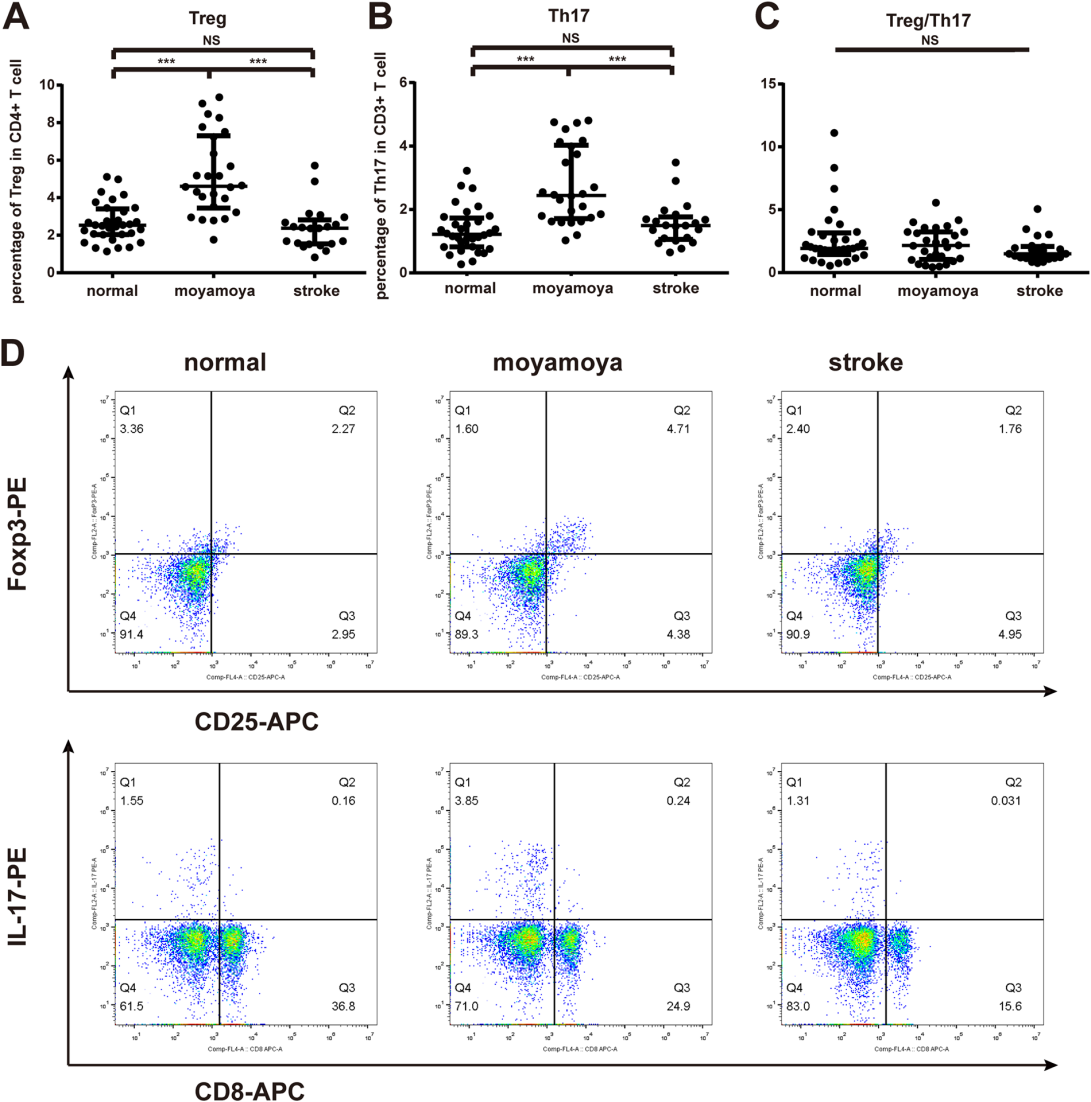
The percentage of peripheral Treg and Th17 cells in MMD patients, atherothrombotic stroke patients and controls. Both the levels of Treg and Th17 were significantly increased in MMD patients compared to other groups. (**A**,**B**) Representative FACS plots for Treg and Th17 cells in peripheral blood. (**C**) The ratio of Treg/Th17 among the three groups. (**D**) Each bar represents the median and quartiles of three independent experiments. *P < 0.05, **P < 0.01 and ***P < 0.001.

To clarify whether imbalance of peripheral Treg/Th17 is involved in the pathophysiology of MMD, the percentage of Treg and Th17 cells from circulating CD4^+^ T cells and their dominantly secreting inflammatory cytokines were measured in MMD patients and control. Results showed that Treg and Th17 were significantly increased in MMD patients [4.615 (3.530, 7.240) and 2.440 (1.740, 3.990)] compared to atherothrombotic stroke [2.370 (1.550, 2.680), *P* < 0.0001 and 1.490 (1.090, 1.690), *P* < 0.0001)] or healthy control group [2.525 (2.040, 3.350), *P* < 0.0001 and 1.215 (0.825, 1.695), *P* < 0.0001] (Fig. 1A,B). However, there was no difference in the ratio of Treg/Th17 among the three groups (*P* = 0.244, Fig. 1C).

In consistent, serum expression of Treg-related TGF-β and IL-10 was enhanced by 121.67% [34.262 (28.016, 45.254) vs. 16.973 (12.117, 19.643), *P* < 0.001] and 8.83% [12.855 (12.210, 13.620) vs. 12.000 (11.155, 12.860), *P* = 0.0029] in MMD patients compared with control. Similarly, Th17-related IL-17, TNF-α, IL-6 and IL-23 were significantly higher than control by 57.94% [130.655 (99.710, 150.030) vs. 78.565 (74.070, 85.455), *P* < 0.001], 7.33% [16.680 (15.870, 17.620) vs. 15.970 (14.500, 16.835), *P* < 0.05], 92.52% [8.420 (7.130, 11.770) vs. 5.285 (2.855, 7.265), *P* < 0.001] and 39.47% [10.925 (8.200, 15.800) vs. 8.185 (6.950, 9.575), *P* < 0.01] in MMD patients, respectively (Supplement Fig. 1). Nevertheless, the level of Th1/Th2-related cytokines, such as IL-2, IL-4, and INF-γ was not significantly different between the two groups [IL-2: 6.860 (5.930, 8.280) vs. 6.660 (5.630, 8.180), *P* = 0.9271; IL-4: 11.210 (9.720, 12.370) vs. 10.560 (9.180, 12.430), *P* = 0.8562; INF-γ: 14.030 (13.580, 15.000) vs. 13.840 (13.160, 14.675), *P* = 0.5483].

### Decreased suppressive function of peripheral Treg in MMD patients

**Figure 2:**
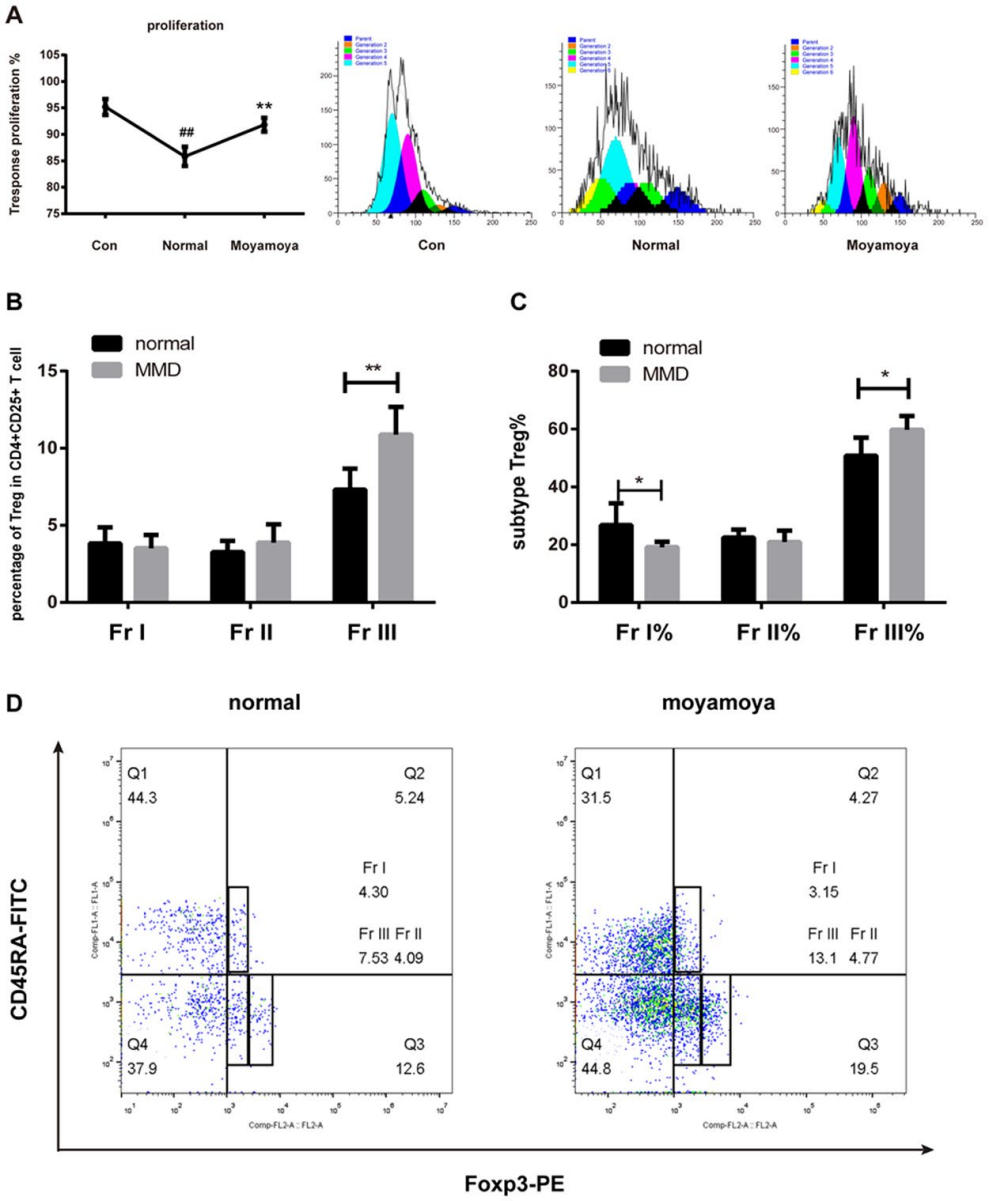
The proliferation of CD4^+^CD25^−^ T cells co-cultured with Treg (**A**). The suppressive function of Treg isolated from MMD patients was lower than controls. The changes of Treg subtypes in MMD patients and healthy controls. Both the percentage of FrIII Treg cells among CD4^+^CD25^+^ T cells (**B**) and total Treg cells (**C**) were significantly elevated in MMD patients. FACS plots for subtypes of Treg cells in MMD patients and healthy controls (**D**). Each bar represents the mean ± SD of three independent experiments. *P < 0.05 and **P < 0.01 vs. normal Treg cells group, ^##^ *P* < 0.01 vs. control group.

Since no difference was found in the ratio of Treg/Th17, we further investigated whether suppressive function of Treg in MMD patients was altered. Interestingly, we found that the Treg from MMD patients had a lower suppressive function compared to those from control using proliferative assay (91.81 ± 0.6439 vs 85.84 ± 0.9120, *P* = 0.0017, Fig. 2A).

Different Treg subsets have been shown to potent distinct functions. We therefore further measured changes in the percentage of three major Treg subtypes. The results showed that the percentage of FrIII Treg cells among CD4^+^CD25^+^ T cells and CD4^+^CD25^+^FoxP3^+^Tregs were significantly elevated in MMD patients compared to control (10.88 ± 0.8052 vs 7.324 ± 0.4782 among CD4^+^, *P* = 0.0018; 59.86 ± 2.095 vs 50.74 ± 2.238 among Treg, *P* = 0.0184, Fig. 2B,C). However, the proportion of FrI Treg among total Treg was markedly decreased in MMD patients (19.17 ± 0.8452 vs 28.37 ± 2.456 among Treg, *P* = 0.0126). Meanwhile, no significant difference was found in the proportion of FrII Treg cells between the two groups (3.876 ± 0.5328 vs 3.269 ± 0.2573 among CD4^+^, *P* = 0.2733; 20.97 ± 1.755 vs 22.52 ± 0.9697 among Treg, *P* = 0.4172). FrIII Treg cells are lack of suppressive functions. These findings suggested that Treg which induced inflammation and lacked suppressive capacity of T cells resulted from increasing FrIII Treg in MMD patients.

### Increased serum expression of VEGF in MMD patients

Moreover, the serum protein expression of angiogenic factor, VEGF was significantly increased by five folds [29.060 (13.910, 63.150) vs. 7.360 (5.325, 10.035), *P* < 0.0001] in MMD patients compared to control, which was consistent with previous findings^16^. Intriguingly, results of the multiple linear regression analysis showed that TGF-β had a correlation with VEGF (P < 0.01, Table 3).

**Table 3:** The correlation of serum VEGF levels with various parameters by linear regression analysis.

	B	S.E.	95% CI	Sig.
Treg	0.632	2.074	−3.53–4.794	0.762
Th17	−2.298	3.332	−8.984–4.388	0.494
TGF-β	0.929	0.277	0.372–1.486	0.002**
IL-10	1.561	2.501	−3.457–6.578	0.535
IL-17	0.088	0.083	−0.079–0.255	0.295

B, coefficients; S.E. : SEM; **P < 0.01.

### Treg and TGF-β is associated with the pathophysiology of MMD

Finally, we investigated the correlation of the inflammatory and angiogenic factors with the severity of MMD. 26 MMD patients were divided into 3 grades based on the Suzuki’s angiographic stage of MMD: grade 1 (Suzuki’s stage 1 and 2, n = 2), grade 2 (Suzuki’s stage 3 and 4, n = 15) and grade 3 (Suzuki’s stage 5 and 6, n = 9). The results suggested that the Suzuki’s angiographic stage is positively correlated with the level of Treg, TGF-β and the ratio of Treg/Th17, but not Th17 and IL-17 (Table 4). To investigate the predictive role of serum Treg and TGF-β for the severity of MMD, binary logistic regression analysis was used. And the results showed that the higher level of Treg could be considered as an predictive factor of (OR 3.842, 95% CI 1.093-13.505) grade 3, compared to grade 2, of MMD after adjusting TGF-β (Table 5). However, these factors had no relationship with onset symptoms and gender.

**Table 4:** Correlations between Treg/Th17 and relevant cytokines and the Suzuki’s angiographic stage.

	Grade 2 (N = 15)	Grade 3 (N = 9)	P value
Treg	3.745 (2.813, 4.598)	5.420 (4.088, 7.443)	0.0068**
Th17	2.170 (1.650, 3.450)	2.450 (1.785, 3.340)	0.7526
TGF-β	31.730 (28.020, 41.470)	48.250 (30.800, 64.190)	0.0112*
IL-17	129.800 (95.800, 146.000)	121.100 (93.390, 153.900)	0.8537
Treg/Th17	1.825 (0.697, 2.222)	2.746 (1.312, 3.132)	0.0401*
IL-10	12.830 (11.900, 14.740)	13.150 (12.490, 13.760)	0.8567
IL-6	8.420 (7.450, 11.770)	8.420 (5.985, 17.350)	0.5466
IL-23	12.280 (7.770, 15.800)	9.650 (7.520, 17.560)	0.9844
TNF-α	16.740 (15.870, 18.500)	16.680 (15.720, 17.310)	0.3745
VEGF	33.450 (13.910, 63.150)	28.760 (17.940, 68.760)	0.7744
ICAM-1	38.600 (31.750, 49.650)	35.350 (26.490, 39.800)	0.1294
VCAM	187.900 (143.000, 222.000)	132.600 (99.470, 210.900)	0.1771
HMGB-1	59.000 (25.500, 106.000)	66.000 (35.000, 185.000)	0.3284

Notes: Correlation is significant at the 0.05 level. *P < 0.05, **P < 0.01.

**Table 5:** The effects of Treg and TGF-βon Suzuki’s angiographic stage by binary logistic regression analysis.

	B	S.E.	Sig.	Exp(B)	95% CI for Exp (B)
Treg	1.346	0.641	0.036*	3.842	1.093–13.505
TGF-β	0.14	0.074	0.059	1.151	0.995–1.331
Constant	−12.153	5.214	0.02	0	

B, coefficients; SE, SEM; Exp(B), OR; *P < 0.05.

## Discussion

Here, for the first time, we reported: 1) Circulating Treg and Th17 cells were higher in MMD patients than that in atherothrombotic stroke and healthy controls. 2) The dominantly expressed FrIII Treg subset in MMD patients may be responsible for the deficient suppressive function of Treg. 3) TGF-β had a positive relationship with VEGF. 4) Treg is an independent factor associated with MMD Suzuki’s angiographic stage.

In the present study, the ratio of Treg/Th17 was not significantly different between MMD patients and control subjects. However, Treg from MMD patients showed deficient suppressive functions. We believed that function imbalance in Treg/Th17 may contribute to the pathophysiology of MMD, since imbalance of Treg/Th17 is involved in several human autoimmune diseases and Treg depletion can evoke autoimmunity^17–20^. Suppressive function of Treg cells have been found to be defective in several other autoimmune diseases such as psoriasis^21^, type 1 diabetes mellitus (DM)^22^ and multiple sclerosis (MS)^23^. Subsequently, we found that increased Tregs were enriched with FrIII Tregs. Accumulating evidence have recently shown that Treg possess high plasticity and can acquire functions of effector-like Th cells, lose their suppressive function but secrete pro-inflammatory cytokines, including IL-17^24, 25^. Three distinct Treg subpopulations with different functions in human FoxP3^+^Tregs have been identified. CD4^+^ CD25^+^ FoxP3^low^ CD45RA^+^Tregs (FrI, stable resting Treg), CD4^+^ CD25^+^ FoxP3^high^ CD45RA^−^Tregs (FrII, activated Treg) and CD4^+^CD25^+^FoxP3^low^CD45RA^−^Tregs cells (FrIII, unstable Treg). The FrI and FrII Treg are suppressive^9^, while FrIII Treg are lack of suppressive functions but possess some features of these “ex-Treg” and may be in the process of transforming into Th17 cells. Therefore, in the present study, the increased FrIII Tregs may be responsible for the decreased suppressive functions of Tregs in MMD patients.

C-reactive protein (CRP) is often used as a clinical marker of acute systemic inflammation. In this research, all blood samples of patients were collected at stable phase after stroke and the plasma concentration of CRP shows no significant difference among MMD, atherothrombotic stroke and control group (data not shown). We further found that both of the Treg/Th17 cells dominantly expressed cytokines including IL-10, TGF-β, IL-17, IL-6 and IL-23 were higher in MMD patients than controls. Moreover, IL-2, IL-4 and INF-γ, which are mainly from Th1/Th2, had no significant differences. They could mediate immune and inflammatory responses in several autoimmune diseases, including rheumatoid arthritis (RA), systemic lupus erythematosus (SLE), type 1 DM, MS, allergy, parasitosis, asthma, *et al*.^26, 27^. It suggests that the immune response of MMD is not completely consistent with SLE, RA, and so on. Treg/Th17-mediated autoimmune responses and inflammation may contribute to the pathogenesis of MMD.

In addition, HMGB-1, a late-acting pro-inflammatory cytokine, plays an important role and shows aberrant high level in chronic inflammatory diseases such as RA and SLE^28^. In this study, MMD patients had a higher serum level of HMGB-1 than controls, which is consistent with patients with chronic inflammatory diseases.

Consistent with previous findings, we also found that the serum level of VEGF in MMD patients was dramatically higher than controls^16, 29^. The increased level of VEGF in MMD may be subjected to upregulated levels of Treg cells and Th17 cells. Treg have been shown to secrete VEGFA in response to hypoxia, which plays a critical role in forming a VEGFA-rich microenvironment and promoting angiogenesis^11^, while IL-17 has been implicated in upregulating VEGF and promoting VEGFR expression^30, 31^. Therefore, Treg/Th17 cells may promote angiogenesis of MMD by upregulating the expression of VEGF. In addition, Treg-produced TGF-β1 can also induce production of VEGF and stimulate subsequent angiogenesis^12^. In our study, the increased TGF-β was associated with the VEGF, which indicated that TGF-β may promote the vascular endothelial cell proliferation leading to abnormal vessels hyperplasia in MMD.

However, there are several limitations in this study. First, this is a preliminary study. The sample size is small. Secondly, TGF-β is not specifically expressed by Treg cells. In our future study, we need enroll more patients. To clarify the relationship of TGF-β and VEGF or MMD stage, level of TGF-β expressed by Treg shouled be measured.

## Summary

In conclusion, our research sheds new light on the involvement of Treg/Th17 cells in the pathogenesis of MMD, thereby providing a potential therapeutic target for treatment of MMD patients. A larger number of patients and control subjects should be recruited in the future studies to validate findings in this study.

## Electronic supplementary material

Supplementary Information
