# Supplementary material for: Association of increased Treg and Th17 with pathogenesis of moyamoya disease
Source: Sci Rep. 2017 Jun 8;7:3071. doi: 10.1038/s41598-017-03278-8 (PMC5465197; doi:10.1038/s41598-017-03278-8)

**Association of increased Treg and Th17 with pathogenesis of moyamoya disease**

Leihua Weng^1,2,*^, Xiang Cao^1,3,*^, Lijuan Han^1,3,*^, Haoran Zhao^1,3^, Shuwei Qiu^1,3^, Yaping Yan^5^, Xiaoying Wang^6^, Xiangyan Chen^7^, Weihong Zheng^2^, Xin Xu^1,3^, Yuanyuan Gao^1,3^, Yan Chen^1,3^, Jie Li^8^, Yongbo Yang^1,3, #^, Yun Xu^1,3,4,#^

**Supplementary Figure.1:** Serum expression of inflammatory cytokines and proangiogenic factors in MMD patients and controls. Protein expression of TGF-β (**A**) and IL-10 (**B**), IL-17 (**E**), TNF-α (**F**), IL-6 (**H**), IL-23 (**I**), HMGB-1 (**J**), VEGF (**K**), ICAM-1 (**L**) and VCAM (**M**) were significantly enhanced in MMD patients compared to controls. However, IL-2 (**C**), IL-4 (**D**) and IFN-γ (**G**) were not. Each bar represents the median and quartiles of three independent experiments. **P* < 0.05, ***P* < 0.01 and ****P*<0.001.


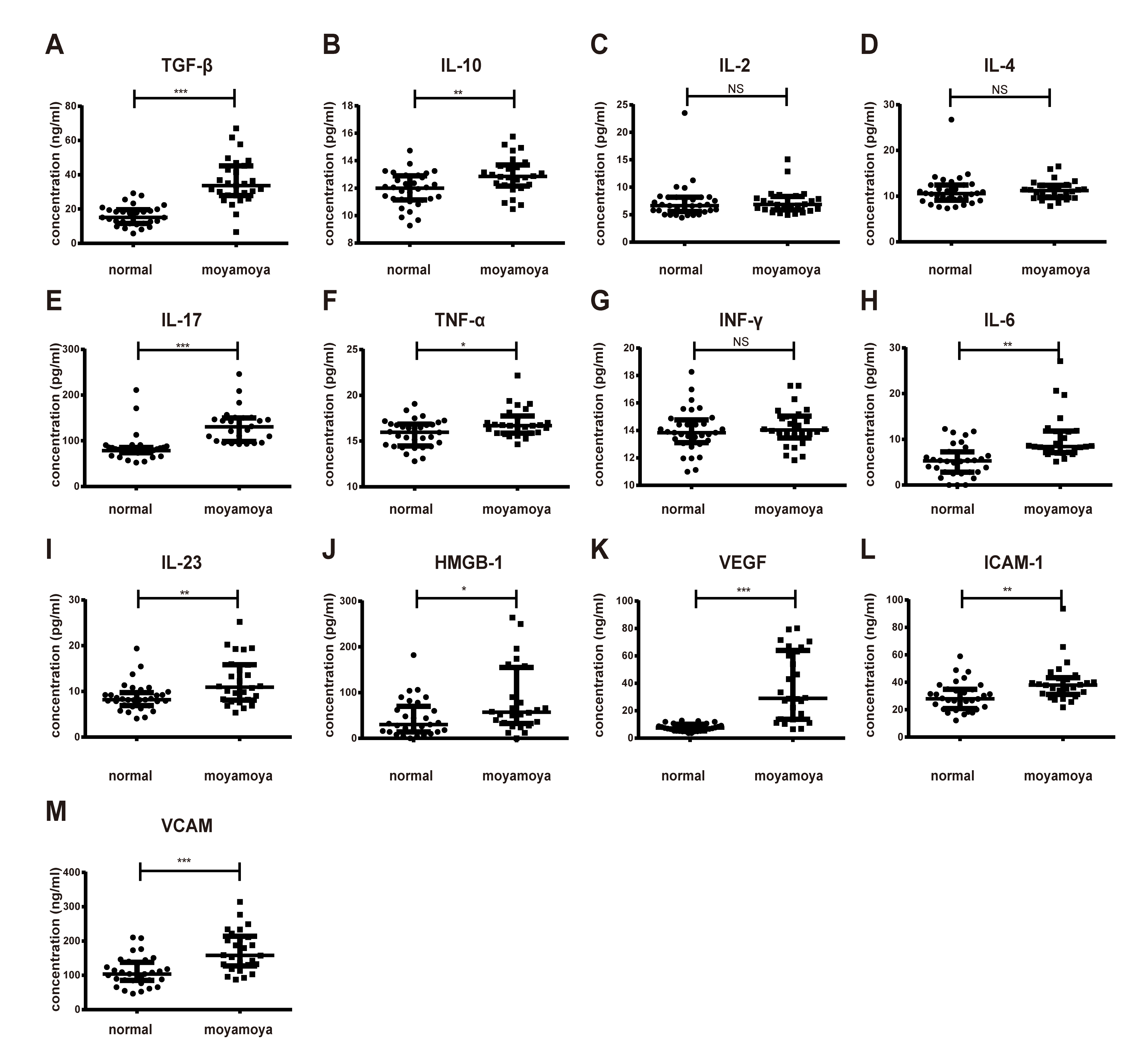

Supplement: Supplementary file 1 — Supplementary Information [file 41598_2017_3278_MOESM1_ESM.doc]
